# Use of Quantitative Dried Blood Spots to Evaluate the Post-Vaccination Level of Neutralizing Antibodies against SARS-CoV-2

**DOI:** 10.3390/life11111125

**Published:** 2021-10-22

**Authors:** Alexandre Marchand, Ingrid Roulland, Florian Semence, Olof Beck, Magnus Ericsson

**Affiliations:** 1 Analysis Department, Agence Française de Lutte Contre le Dopage (AFLD), 143 Avenue Roger Salengro, 92290 Châtenay-Malabry, France; I.roulland@afld.fr (I.R.); F.semence@afld.fr (F.S.); M.ericsson@afld.fr (M.E.); 2 Department of Clinical Neuroscience, Karolinska Institute, 171 77 Stockholm, Sweden; olof.beck@ki.se

**Keywords:** vaccination against SARS-CoV-2, neutralizing antibodies detection, quantitative dried blood spots, plasma, immune status

## Abstract

To combat the COVID-19 pandemic, vaccines against SARS-CoV-2 are now given to protect populations worldwide. The level of neutralizing antibodies following the vaccination will evolve with time and vary between individuals. Immunoassays quantifying immunoglobulins against the viral spike (S) protein in serum/plasma have been developed, but the need for venous blood samples could limit the frequency and scale of control in populations. The use of a quantitative dried blood spot (DBS) that can be self-collected would simplify this monitoring. The objective of this study was to determine whether a quantitative DBS device (Capitainer qDBS 10 µL) could be used in combination with an Elecsys anti-SARS-CoV-2 S immunoassay from Roche to follow the development and persistence of anti-S antibodies. This objective was carried out through two clinical studies. The first study investigated 14 volunteers who received two doses of the Comirnaty (Pfizer) vaccine. The levels of anti-S antibodies and the progression over time post-vaccination were studied for three months. The level of produced antibodies varied between subjects, but a similar trend was observed. The anti-S antibodies were highly stimulated by the second dose (×100) and peaked two weeks later. The antibody levels subsequently decreased and three months later were down to 65%. DBS proved to be sufficiently sensitive for use in evaluating the immune status against SARS-CoV-2 over a prolonged time. The second cohort was composed of 200 random patients from a clinical chemistry department in Stockholm. In this cohort, we had no information on previous COVID-19 infections or vaccination. Nevertheless, 87% of the subjects had anti-S immunoglobulins over 0.8 U/mL, and the bias between plasma and DBS proved to be variable, as was also seen in the first vaccination study.

## 1. Introduction

Severe acute respiratory syndrome coronavirus 2 (SARS-CoV-2) emerged in 2019 as a new highly infectious virus responsible for coronavirus disease 2019 (COVID-19) and caused a worldwide pandemic, the most severe since the Spanish flu a century ago. A wide variety of symptoms can be observed after infection, including mild to severe pneumonia, extreme fatigue, and peculiar symptoms like loss of taste and smell. More than 200 million people have been diagnosed as positive for COVID-19, and the number of deaths directly caused by the infection exceeds 4 million worldwide. Pharmaceutical companies reacted quickly to the rising pandemic, and several vaccines were proposed, clinically tested, and approved for use in the general population within two years. These vaccines are now used worldwide. A new kind of vaccine proved to be very efficient against the virus: two mRNA vaccines, Comirnaty (Pfizer/BioNtech) and Spikevax (Moderna) [1,2]. With these vaccines, mRNA encoding the SARS-CoV-2 viral spike (S) protein, which plays an essential role in viral infectivity, is introduced into human cells. The viral protein is then produced by the human cells and released into the blood, triggering an immune response and the production of antibodies. In the case of subsequent infection by SARS-CoV-2, the anti-S antibodies already present in the blood will directly recognize the S protein on the surface of the virus and neutralize the virus, limiting its capacity to infect cells and spread in the human body. Vaccination now seems to be the best way to limit the severity of syndromes, reproduction of the virus, and transmission to other people. However, the duration of protection post-vaccination is still unknown and could depend on the persistence of these neutralizing antibodies.

Neutralizing antibodies are also produced during the course of SARS-CoV-2 infection, generally within the first three weeks of symptom onset. The early presence of these antibodies is positively correlated with the survival of hospitalized patients [3]. Neutralizing antibody titers rise for a few weeks until the progression of the viral infection is controlled, and then they progressively decrease. Dispirensi et al. showed that anti-S IgG was highly correlated with neutralizing activity and that its presence was long-lasting, persisting for up to 8 months in most recovered patients [4]. However, the longevity of immunity will vary among individuals, and a small proportion of the population will not produce/keep neutralizing antibodies, with rapid waning, slow waning, or persistent neutralizing antibodies observed within six months post-infection [5]. It remains to be clearly determined whether people re-infected with SARS-CoV-2 have low to no remaining levels of neutralizing antibodies or their antibodies are less susceptible to neutralizing viral variants [6]. In any case, plasma from recovered COVID-19 patients with high neutralizing antibody titers has shown therapeutic effects when transfused to patients with severe COVID-19, and this potential therapeutic strategy is under investigation [7].

With many people who are asymptomatic or have mild symptoms, especially among the young, it can be difficult to obtain a realistic estimate of the prevalence of SARS-CoV-2 infections, and the detection of long-lasting anti-S IgG could be used to better evaluate the diffusion of the virus in the populations of various regions/countries. In addition, it might be important to know whether anti-SARS-CoV-2 antibodies are present prior to vaccination since the antibody response to SARS-CoV-2 vaccination can produce very high titers in previously infected people, even those who were asymptomatic. This could increase the risk of adverse effects after the second dose and has thus prompted the recommendation of a single dose for those previously infected [8]. With the long-lasting pandemic and the emergence of new variants, a third dose of vaccine might be necessary after six months, especially if people have low levels of remaining neutralizing antibodies.

Rapidly and accurately quantifying anti-S Ig in blood would be helpful in all these situations and would provide valuable data for taking appropriate measures regarding each individual situation. Dozens of immunoassays have been developed to test SARS-CoV-2 antibodies, from classic ELISA to lateral flow immunoassays [9] and automated chemiluminescent assays [10]. Due to their high standardization and the worldwide diffusion of analyzers for clinical chemistry tests, the use of automated chemiluminescent assays appears to be the most attractive option. As with other clinical assays for circulating proteins, these assays are validated on serum/plasma. This can limit the large-scale testing of populations and longitudinal studies because trained phlebotomists are needed to perform venipuncture to acquire samples. A frequent alternative to simplify the collection of samples is the use of a dried blood spot (DBS) obtained by a finger prick. A finger prick can be performed by the patient or those with little training at home or in the field. Following sample collection, the collection cards are then be sent to a laboratory using regular mail. The dried blood stabilizes many analytes, including immunoglobulins IgG, IgM, and IgA, and it was shown that Ig quantification from DBS was not affected after 14 days of storage at room temperature [11]. However, hematocrit variations and variability in the collected blood volume can add variability to the process. Therefore, volumetric/quantitative DBS devices have been developed in recent years. These devices have overcome these sources of variability [12] and added more reliability to the results.

The objective of this study was to determine whether a quantitative DBS device, the Capitainer qDBS, which collects a precise 10-µL volume on a paper spot independently of the initial volume of the blood drop, could be used to measure anti-S antibody concentration in blood with the automated Elecsys anti-SARS-CoV-2 S immunoassay from Roche. This would simplify the evaluation of the immunity status against SARS-CoV-2 in large populations. After validating the conditions to perform the dosage from DBS, the development of the immune response after vaccination and the persistence of the anti-S antibodies was first evaluated in a cohort of 14 volunteers. These subjects were followed regularly from prior to vaccination up to three months following the second dose of the Comirnaty vaccine. Changes in the anti-S antibody concentrations in blood detected in the plasma samples and auto-collected qDBS samples were compared throughout the study. Second, plasma and DBS samples were obtained from a cohort of 200 volunteers and analyzed, with the results then compared to further investigate the bias in concentration level between plasma and DBS. These volunteers were random patients at a clinical chemistry department in the Stockholm region.

## 2. Material and Methods

### 2.1. Blood Samples

Human volunteers were recruited before they were vaccinated against SARS-CoV-2. They had not tested positive for SARS-CoV-2 prior to vaccination except for one person. All participants signed a written consent and authorization to collect and store their blood samples, which was approved by the bioethics committee of the French *Ministère de l’Education nationale, de l’Enseignement supérieur et de la Recherche* with registration number DC-2019-3644. For analysis on plasma, venous blood was collected in K2 EDTA tubes (BD Vacutainer, Plymouth, UK) by a phlebotomist. Blood tubes were centrifuged for 10 min at 1500× *g* to separate the plasma from the blood cell pellets and the tubes were stored at 4 °C until analysis. For DBS, self-collection was performed by each volunteer using a BD Microtainer^®^ Contact-Activated Lancet Blade (1.5 mm × 2.0 mm) for the finger prick and collected on a Capitainer qDBS 2 spot card (Capitainer AB, Stockholm, Sweden) following the manufacturer’s instructions. Two circular paper spots of 10 µL were obtained. After a couple of hours to let the blood dry, the cards were stored at room temperature in a box with desiccant until analysis.

A second cohort of 200 random volunteers from Stockholm’s general population was recruited by the Clinical Chemistry Department of the Karolinska University Hospital after authorization from the Swedish Ethical Review Authority (Dnr 2020-04219) to perform plasma/DBS collection to evaluate anti-SARS-CoV-2 antibodies. An aliquot of venous EDTA-blood sample collected by a phlebotomist was used to prepare parallel DBS and plasma samples from each volunteer [13].

### 2.2. Sample Preparation

For plasma analysis: 120 µL of plasma was directly pipetted from the centrifuged blood tubes in 2 mL Sample Cups (Roche Diagnostics, Meylan, France) directly placed on the analyzer.

For DBS, immunoglobulins were first extracted from one spot of the qDBS Capitainer card: a 10 µL spot was removed using tweezers and put into a 2 mL Protein LoBind^®^ tube (Eppendorf, Montesson, France). Then, 150 µL of PBS (phosphate-buffered saline pH 7.4, prepared with PBS Tablets, Interchim, Montluçon, France) with 0.5% Tween-20 (Merck, Saint-Quentin Fallavier, France) were added to the tube. Agitation was performed at room temperature on a thermomixer (Eppendorf France, Montesson, France) at 450 rpm for 1 h 30 min. Next, 120 µL were pipetted into 2 mL Sample Cups and directly analyzed.

### 2.3. SARS-CoV-2 S Immunoassay

Elecsys^®^ Anti-SARS-CoV-2 S immunoassay (Roche Diagnostics) is an automatized electrochemiluminescence immunoassay (ECLIA) using a double-antigen sandwich assay format, validated for the quantitative detection of high-affinity antibodies (including IgG) against the SARS-CoV-2 virus in serum and plasma [14]. The assay used the receptor-binding domain (RBD) part of the SARS-CoV-2 spike protein (S) as an antigen for the capture of anti-S antibodies, including long-lasting IgG, which is considered to be the main neutralizing antibody against SARS-CoV-2 [15]. It was analyzed on a Cobas e411 Analyzer (Roche Diagnostics, Meylan, France) following the manufacturer’s recommendations using dedicated calibrators (calibration was performed once a week) and negative and positive controls (added to each run with the samples). The time to conduct the analysis and obtain the result for one sample was 18 min. Numeric values were directly obtained within the linear range of 0.4−250 U/mL, and a result “<0.4” (the limit of detection: LOD of the test) was considered equal to 0 and “>250” required additional dilution of the sample with Roche Multi-Assay diluent for Cobas (DIL MA) to obtain a value within the linear range when a precise quantification was needed.

### 2.4. Data Interpretation

With the Elecsys^®^ Anti-SARS-CoV-2 S assay, 1 nM of monoclonal anti-SARS-CoV-2 S antibodies (internal Roche standard) corresponded to 20 U/mL. According to Roche [14], a sufficient concentration of antibodies to postulate neutralization in the serum/plasma in case of infection with the SARS-CoV-2 is 0.8 U/mL, which was adopted as the decision limit (<0.8 U/mL = non-reactive, ≥0.8 U/mL = reactive). This was established based on considering values obtained in samples of patients 14 days following a positive PCR test for COVID-19 (when a humoral response has taken place) and was validated in a comparison study with the results from a VSV-based pseudo-neutralization assay [16,17].

### 2.5. Adaptation of the Elecsys^®^ Anti-SARS-CoV-2 S Immunoassay to DBS

To test the conditions for analysis with DBS, four controls were prepared by spiking the whole blood of a subject with no detectable anti-S antibodies with different amounts of plasma from a recovered COVID-19 patient with high levels of anti-S antibodies (690 U/mL). Capitainer qDBS cards were spotted with the spiked blood, and the plasmas were isolated and analyzed (C1: 14.8 U/mL, C2: 7.5 U/mL, C3: 129.1 U/mL, C4: 59.8 U/mL). Different conditions of desorption of immunoglobulins from the DBS were tested: buffer (PBS, PBS-T-0.5%, PBS-T 1%), time of desorption (overnight or 90 min), and sonication (15 min vs. no step). The condition that gave the best detection rate was selected for all further experiments (see Sample Preparation). Limit of quantification (LOQ), reproducibility, and analyte stability was determined by analyzing the DBS controls in quadruplicates on 5 different days over 2 weeks).

### 2.6. Clinical Study Performed to Follow the Development and Stability of the Antibodies Produced after Vaccination against SARS-CoV-2

The subjects were 9 males and 6 females, healthy with no chronic disease/medication, and 28–55 years old. They were tested prior to the primary vaccination and then regularly in between and after their second vaccination (Comirnaty, Pfizer-BioNTech). DBS and plasma were collected starting 2 weeks after the first dose and then once a week for DBS and every 2 weeks for plasma. The second dose of vaccine was given 6 weeks after the first dose, and sample collection continued for 3 months. All subjects completed the entire protocol, but due to vacations, some missed 1–2 sampling times, particularly for the plasma sample, although most continued to self-collect DBS even during their vacations.

## 3. Results

To evaluate the detection of anti-S antibodies from DBS in the volunteers, we first validated the protocol of desorption from the DBS. Various conditions were tested to find a combination between a high-extraction recovery and a protocol with few steps. Starting from an in-house protocol previously used to test antibodies against the nucleocapsid of the SARS-CoV-2 [18], various optimization steps were tested according to other published papers [19,20,21]. Adding Tween-20 (up to 1%) to PBS buffer slightly increased the measured concentrations, and PBS + T 0.5% was the final buffer selected. Adding a 15-min sonication step did not produce any increase and was not adopted. To shorten the overall time of analysis, the desorption step was decreased from overnight to 90 min without loss in extraction recovery. The final buffer volume adopted in these experiments was 150 µL of PBS-T 0.5% to ensure 120 µL for the assay even in cases of slight retention of buffer by the paper spot. The minimal working volume was indeed 120 µL with the Sample Cups adapted to the Cobas model e411 used for analysis. With this protocol, the controls spiked with various levels of anti-S antibodies showed that one 10-µL spot gave results 35–45 fold lower than the ones measured in plasma independently of the initial plasma value, which mainly reflected the dilution effect. Control C1 spotted on DBS was just at the LOD of the analyzer (0.4 U/mL) and corresponded to 14.8 U/mL in plasma, which could be considered the corresponding limit of quantification when the analysis was performed from a DBS. The DBS gave reproducible results with an intra-day coefficient of variation (CV%) below 5% and an inter-day CV% below 10% (Table 1). However, a 10% decrease was observed after 2 weeks compared to an analysis performed the day following the sample collection. A 2-week window seemed appropriate to analyze the samples in a clinical context, so this was considered acceptable. All DBS analyses from this post-vaccination study were performed less than a week after sample collection.

**Figure 1:**
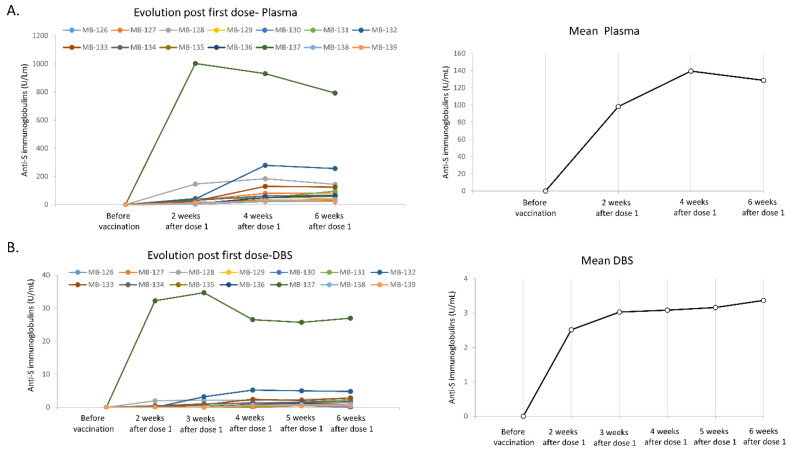
Change in anti-S immunoglobulins measured in plasma and DBS before and up to 6 weeks after the first dose of the Comirnaty vaccine. All 14 volunteers’ (MB-126 to MB-134) values measured in plasma part (**A**) and DBS part (**B**) with Elecsys Anti-SARS-CoV-2 S assay are presented up to six weeks after the first vaccine dose (just before the second dose). Volunteers were tested every two weeks with plasma and once a week with DBS. The mean value of the anti-S determined in the cohort at each time is also presented on the left.

To study the development of the levels of neutralizing antibodies under the vaccination scheme and their post-vaccination changes, a phlebotomist collected a tube of whole blood (plasma) every 2 weeks from each subject, while the Capitainer qDBS was auto-collected following the manufacturer’s instructions after a finger prick every week. Only one spot from the Capitainer card was analyzed each time. The second one was only used if a new analysis needed to be performed to confirm an atypical data item or an analytical problem arose with the first one. This was nevertheless rare. None of the subjects had any detectable anti-S Ig before vaccination, with all values from plasma and serum being below 0.4 U/mL. Two weeks after the first dose of the Comirnaty vaccine, all subjects had produced neutralizing antibodies and plasma anti-S Ig concentrations were over 0.8 U/mL, which defined a reactive plasma with neutralizing activities (Figure 1 and Table 2). However, there were differences between the subjects. The level of anti-S Ig ranged from 2.69 to 1003 U/mL (median 20 U/mL). DBS values were below the LOD (<0.4 U/mL) or just above for most subjects, and only two of these subjects, those with the highest plasma values, had corresponding DBS values above the 0.8 U/mL threshold. Two weeks later, the anti-S antibodies titers showed a rise for the subjects who had previously shown low levels of antibodies, and DBS analysis showed results over the LOD for all subjects except one. As expected, every DBS value was between 30- and 90-fold lower than the corresponding plasma values. The fold change between plasma and DBS was variable, possibly due to the low level of antibodies in the DBS leading to increased measurement uncertainty at values close to the LOD. However, any DBS result obtained over the LOD of the assay (0.4 U/mL) clearly indicated a reactive plasma. The level of antibodies was then mostly stable (slightly decreased/increased depending on the subject) at 6 weeks post-vaccination, just before the second dose. All 14 subjects had a similar evolution. Only one volunteer’s result was below the LOD with the DBS analysis 6 weeks post-vaccination, and notably, it was also the one with the lowest corresponding plasma value (21.4 U/mL).

**Figure 2:**
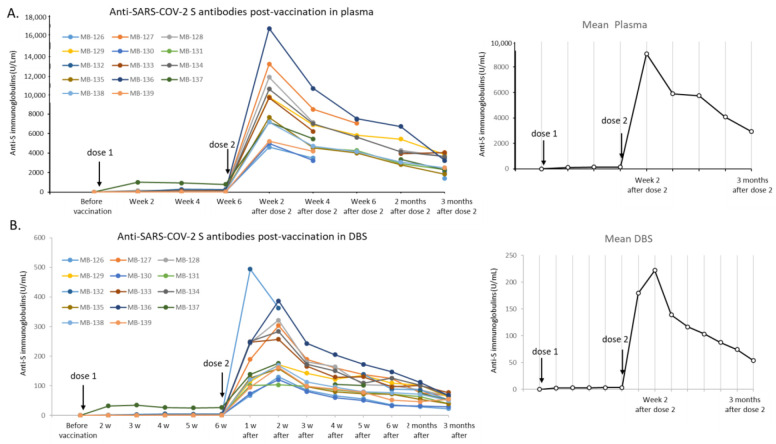
Change in anti-S immunoglobulins measured in plasma and DBS before, after dose one, and after dose two of the Comirnaty vaccine, with a three-month follow-up. All 14 volunteers’ (MB-126 to MB-134) values measured in plasma part (**A**) and DBS part (**B**) with Elecsys Anti-SARS-CoV-2 S assay are presented up to three months after the final dose of vaccine. Some volunteers missed sampling times, but all terminated the study with the final time-point three months after the second dose of vaccine. The mean value of the anti-S immunoglobulins determined in the cohort at each time is also presented on the left.

The second vaccine dose was administered to all volunteers six weeks after the first dose. There was more than a 100-fold rise in the antibody titers detected one week following the second dose (Table 3 and Figure 2). Anti-S Ig reached its maximum blood concentration the second week after the second dose, on an average 188 times for DBS and 175 times for plasma anti-S Ig compared to the concentrations before the second injection. One subject had a relatively low increase compared to the other subjects (MB-137, 6.5 times). However, this subject had already high levels of anti-S antibodies after the first dose, approximately 10 times higher than the average for the rest of the cohort. All the volunteers had anti-S Ig titers between 4500 and 13,000 U/mL in plasma, which were all far above the linear range of the Elecsys immunoassay (all results were >250 U/mL). To obtain a value within the linear range of the method, plasma dilution to 1/50 to 1/100 was necessary, and a new analysis was subsequently performed. The analysis of the DBS, on the contrary, gave mainly values within the linear range due to the approximate 50-fold difference compared to plasma levels (results 103.6 to 387.2). Hence, even though more laborious, using DBS instead of plasma simplified the analysis because the need for the dilution step was reduced. The anti-S Ig titers measured by DBS were decreased after one week (−38%), which was confirmed a week later in plasma by a drop of 35% compared to the maximal levels observed two weeks before. The antibody titers continued to drop in the following weeks. Two months after the second dose, the antibody levels were decreased by 55% in plasma and 61.5% in DBS and three months later by 68% in plasma and 76% in DBS compared to the maximum reached earlier (meaning a 3–4-fold decrease in 2.5 months). All subjects showed a similar trend in decrease. However, despite this decrease, the anti-S titers in the samples were still above the 0.8 threshold for a reactive plasma: 1418 to 4055 U/mL in plasma, and 22.6 to 77.6 U/mL in DBS, still 50-fold over the LOD.

**Figure 3:**
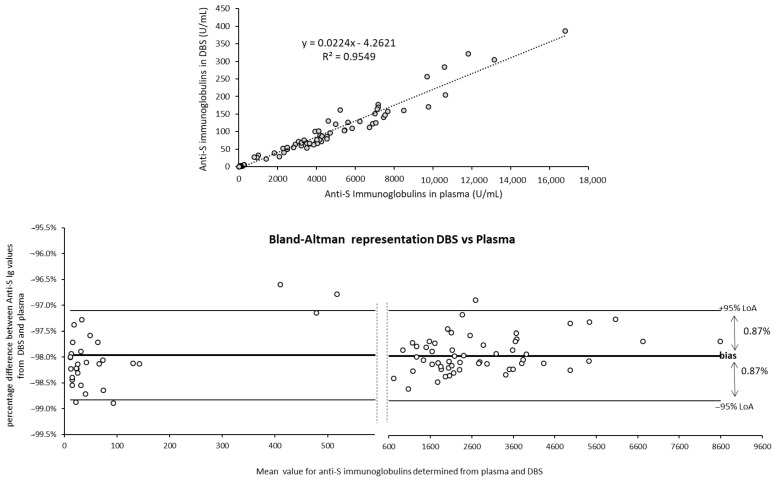
Comparison of Elecsys^®^ Anti-SARS-CoV-2 S values obtained with plasma and qDBS for the 14 volunteers during the five-month study. A: Graphic representations of all samples for which a detectable anti-S Ig value was obtained in plasma and DBS for the 14 volunteers during the five-month study (*n* = 86). Linear regression is represented by a dotted line; equation and coefficient of determination are indicated. B: Bland-Altman plot (percentage of difference DBS-plasma/mean of values DBS-plasma). Mean bias (%) is indicated by a bold line; 95% limits of agreement (LoA) are shown by thin lines.

Throughout the study, quite a similar fold change was observed between plasma and DBS values for all subjects: approximately 50-fold. The correlation between plasma and DBS values was evaluated, taking into account all the data (Figure 3), and a coefficient of determination R^2^ = 0.955 was observed for linear regression. Concordantly, a Bland-Altman plot showed that the vast majority of the values (94.2%, 81/86), from low to high titers, were within a tight 95% confidence interval (1.75%) but with a mean bias of −98.0% between DBS and plasma. This indicated a 49-fold change. If this factor was applied to all the DBS values, the mean bias between DBS and plasma values would be <1.5%, confirming that the two matrices were in agreement despite the difference in sensitivity. 

This DBS method was also evaluated in subjects receiving other types of vaccines (AstraZeneca, Janssen) and in a subject who had been hospitalized after COVID-19 infection and then vaccinated with Comirnaty. The results (Table 4) confirmed the potential to use DBS to quantify anti-S IgG I in people who had received any kind of vaccination. All showed levels of antibodies above the threshold, especially the previously mentioned COVID-19 patient who had received two doses of vaccine. All levels were detectable using DBS.

**Figure 4:**
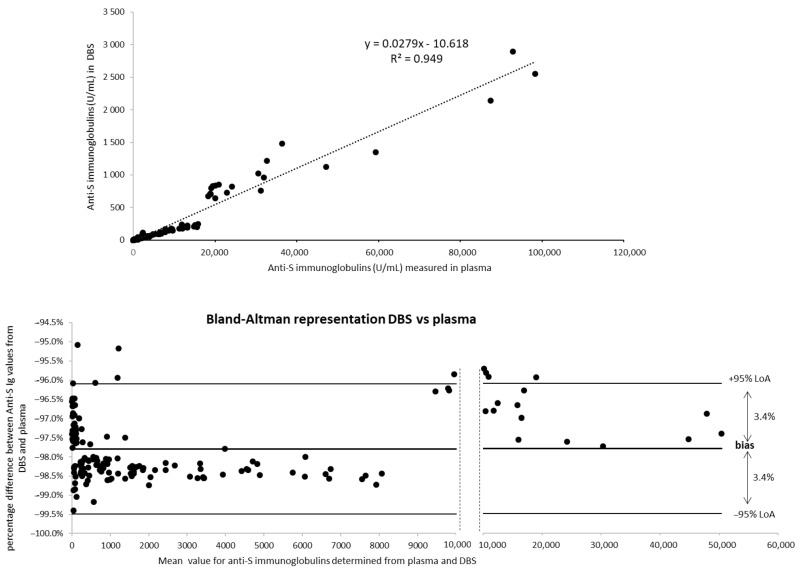
Comparison of Elecsys^®^ Anti-SARS-CoV-2 S values obtained with plasma and qDBS for the second cohort of volunteers (single-time analysis). A: Graphic representations of data from 159 volunteers with an anti-S Ig titer obtained in plasma and DBS. Linear regression is represented by a dotted line; equation and coefficient of determination are indicated. B: Bland-Altman plot (percentage of difference DBS-plasma/mean of values DBS-plasma). Mean bias (%) is indicated by a bold line; 95% limits of agreement (LoA) are shown by thin lines.

DBS testing to determine the level of anti-S antibodies in the population was also evaluated in the second cohort of 200 volunteers enrolled in August 2021 in Stockholm, Sweden. Volunteers were 60% females from 7 to 97 years old (mean age 54.6 +/− 23.3) and 40% males from 13 to 94 years old (mean age 52.9 +/− 21.1). In this blinded evaluation, no information on previous COVID-19 infection or vaccination was available. As shown in Table 5, 174 subjects (87%) had anti-S immunoglobulins over 0.8 U/mL. Titers were between 1.1 and 98,290 U/mL. As a majority of the volunteers showed high plasma titers, initial screening led to 116 values over the assay range (58%). A new analysis was performed after a 1/100 dilution, but eight samples were still over the range and required a new dilution (1/1000) to obtain a trustworthy value. DBS samples gave mostly values ≥0.4 showing the presence of anti-S immunoglobulins for 159 volunteers (79.5%), and only six samples required a second analysis (1/100 dilution). Titers were between 0.46 and 2896 U/mL. Due to the difference in sensitivity, 15 subjects (7.5%) had anti-S antibodies present in plasma over the threshold but not detected by DBS, but these were subjects with low plasma values (all were below 35 U/mL except one at 126 U/mL). No subject was detected with anti-S antibodies in DBS but not in plasma. The mean fold change between plasma and DBS was 51.3 but with more variability than in the previous cohort (fold changes from 26.9 to 168 were observed, see Table 5). A second DBS spot was analyzed for the 10 samples for which the plasma/DBS fold change was below 30 or over 80 and gave similar results (data not shown). This variability for a small portion of the samples is unexplained. When DBS and plasma values were compared for the samples that had a numeric value with both matrices (*n* = 159), a coefficient of determination R^2^ = 0.949 was observed for linear regression (Figure 4). Concordantly, a Bland-Altman plot showed that the vast majority of the values (94.3%, 150/159), from low to high titers, were within a tight 95% confidence interval (6.8%) but with a mean bias of −97.8% between DBS and plasma (Figure 4). These results agreed with the results from the vaccinated cohort showing that quantitative DBS can be applied as an alternative to plasma to determine anti-S immunoglobulins.

## 4. Discussion

In this study, we showed the possibility of starting from a 10 µL DBS instead of serum/plasma to reliably quantify anti-S immunoglobulins by an automated immunoassay. The preparation step was reduced to a simple desorption and was finished in less than two hours, followed by direct analysis on a Cobas Analyzer. The DBS results were comparable to the plasma results but with an approximately 50-fold difference. This fold difference was relatively stable over a huge range of plasma values, from 20 to 20,000 U/mL, with more variability observed for low plasma values (close to the LOD) or very high values (needing an additional dilution step, potentially adding more variations). This difference between plasma and DBS is mainly due to the initial use of blood instead of plasma/serum and to the desorption step causing dilution in 150 µL buffer to obtain enough volume to perform the analysis on the Cobas e411 analyzer. This impacts the sensitivity of the test, and a low level of anti-S Ig (below 20 U/mL in plasma) will be below the LOD for DBS. However, the sensitivity in our working conditions was sufficient to detect the presence of anti-S antibodies in all the subjects tested after their full vaccination for at least three months and, given the rate of decrease, antibodies should be detectable over the LOD using DBS 9 to 12 months after the final vaccination. In addition, the subjects tested after vaccination with other vaccines all had detectable anti-S antibodies with DBS, and the cohort of 200 subjects with unknown status showed that DBS was sufficiently sensitive to detect 91.3% of the subjects with plasma anti-S levels over 0.8 U/mL and 100% of the subjects with anti-S levels >130 U/mL, far below the titers measured even three months after vaccination. This validates the feasibility of screening a population to evaluate the presence of antibodies, as well as the need for a booster dose of vaccine.

If necessary, the sensitivity of DBS analysis with the Elecsys anti-SARS-CoV-2 S assay could be improved by working with other instruments to enable the use of lower volumes. Recent work showed that a 60 µL volume was sufficient with a Cobas e801 to work with DBS [22]. The initial volume of dried blood could also be increased by using other quantitative/volumetric DBS specimens allowing collection of 20 µL or 30 µL per spot (e.g., Tasso M-20 from TASSO Inc., Mitra from Neoteryx).

To shorten the analytical procedure, the time for desorption of the antibodies from DBS was decreased in our experiments from overnight to 90 min; however, a 10% decrease in the values was observed after two weeks of storage. This might reflect harder desorption from DBS or a small degradation in the specific anti-S antibodies detected, but it would not greatly impact the evaluation of the immune status, which would normally be easily performed within the two-week post-sampling period.

The level of anti-S antibodies post-vaccination was higher than the limit validated by Roche for a reactive serum/plasma (0.8 U/mL), and the plasma sometimes needed several dilutions to be within the range of the assay, contrary to DBS, which most of the time directly gave a value within the assay range. Thus, DBS could be used as an initial testing strategy to evaluate whether neutralizing antibodies are present prior to vaccination in the case of a previous infection, even asymptomatic; after the vaccination, a process to confirm that immunity has developed; and after a few months to see whether antibodies are still present. In the case of undetectable antibodies post-vaccination or SARS-CoV-2 infection by DBS analysis, further investigations would require a venous blood sample to confirm this result, and doing so would identify those people who are at risk of a new SARS-CoV-2 infection. As previously demonstrated in other studies [19,21,23,24,25], our data confirmed that when the analytical process is performed properly, the observed results for DBS and serum/plasma anti-SARS-CoV-2 are in agreement.

Self-collection for DBS could be done with kits bought in a pharmacy or sent to the home, and the need for a complementary venous blood sample would only be necessary for DBS analysis results below the LOD of the assay when immunity is expected. DBS by finger prick is well tolerated, and auto-sampling worked well with our cohort of volunteers. Beyerl et al. performed high-scale monitoring of anti-SARS-CoV-2 antibodies in volunteers with DBS and reported that 4465/4471 of unsupervised home sampling DBS cards (99.87%) could be analyzed [22], thus showing that it is a viable strategy to obtain samples even from untrained individuals. The DBS device used in this study was also successfully used in a large test of auto-sample collection evaluation in Stockholm (878 individual Capitainer cards). In this study, 82% of the participating untrained volunteers successfully sent back a sample that could be analyzed for anti- SARS-CoV-2 antibodies [13]. The advantage of using a quantitative DBS device is that it avoids the bias produced by variations in both hematocrit and the collected blood volume, which might affect the results. Thus, quantitative DBS enables more standardization in the data, which is better for intra- and inter-individual follow-up.

Among many immunoassays (ELISA, lateral flow test, etc.) available to test for neutralizing antibodies, the automated immunoassays are well suited for high-throughput, limiting both the time needed for manual manipulation and the number of technicians needed for a large series of samples. In addition, these analyzers are already present in many clinics and hospitals worldwide for diagnostic tests. The Roche Elecsys anti-SARS-CoV-2 S assay was also demonstrated to be a reliable assay to confirm recent COVID infections [26] and was proposed as a useful tool to evaluate the need for one or two vaccine doses in the case of pre-existing anti-SARS-CoV-2 antibodies, the purpose being to limit potential adverse effects [27]. Higher titers are indeed detected in people with a previous SARS-CoV-2 infection and also those who have been vaccinated compared to people without previous exposure to the virus: a median value of 30,527 U/mL in plasma for recovered COVID-19 patients versus a median value of 1975 U/mL in COVID-19 negative people 10 days post-vaccination [8]. While limited in number, our own observations seem to confirm these observations. Other assays that quantify anti-S antibodies could be used for the same purpose. A recent comparison of Roche Elecsys anti-SARS-CoV-2 S with four other CE-marked automated tests showed a good correlation between assays even if they were not directly comparable in the final concentrations [28].

The dynamics of the post-vaccination immune response showed that the second dose of the Comirnaty vaccine was essential to boost the first antibody production of the first dose. However, there was already a 50% decrease in the antibody titers one month after the peak. This is in good agreement with previous observations of a −1.1% decrease per day after the peak post-second dose in subjects vaccinated with Comirnaty [29]. The evolution in all our subjects was very similar, but they were also all healthy middle-aged adults with no chronic disease. The immune response appeared lower and more prone to fade away in the elderly, who are also the most at risk of developing severe forms of COVID-19 [30]. As the duration of each individual immune status is not easy to extrapolate, frequent testing might be needed, for which the DBS approach is better suited, especially in individuals with fragile health. After several months, the neutralizing antibodies might be too low to guarantee protection against a new contact with the virus in some vaccinated people, especially the immunocompromised and elderly, and a new dose of vaccine is, in that case, recommended to again boost defenses [31,32]. In addition, the problem of rapid mutations leading to emerging variants with potential resistance to the current neutralizing antibodies poses a threat that will probably make repeated vaccinations necessary or prompt the development of new vaccines adapted to specific variants as necessary [33]. Screening for antibody response to specific SARS-CoV-2 antigens after vaccination might be required in the future and could be performed with DBS and automated immunoassays. 

One important question that cannot easily be answered concerns the biological meaning of the anti-S antibodies differences at the individual level over time and how they impact immunity against SARS-CoV-2. The optimal antibody level that correlates with protection is still unclear and might vary among individuals. Data collected by Wang et al. [34] showed that eight weeks after the second injection of anti-SARS-CoV-2 mRNA vaccine, volunteers had significantly higher levels of anti-S IgG and IgM than a cohort of patients who had recovered from COVID-19 (assayed at 1.3 and 6.2 months after infection). The neutralizing activity range observed 3−14 weeks after the second dose was similar to that observed in the recovered patients after 1.3 months and greater than that in the recovered patients 6.2 months after infection. However, neutralizing potency was decreased against some new SARS-CoV-2 variants with mutations on the spike protein. Tretyn et al. [35] studied the humoral immune response in 477 individuals after one or two doses of mRNA vaccine in healthy and recovering COVID-19 patients. They concluded that the humoral immune response was diversified and visible as early as two to three weeks after the first dose of the mRNA vaccine, while the level of protection significantly increased after the second dose. This increase was much greater in pre-vaccine healthy subjects and less in convalescents. Larger studies of different groups of individuals (age, ethnicity, with or without disease or comorbidities, etc.) are needed to precisely determine the duration of the neutralizing activity elicited by the vaccine and the minimal quantity of antibodies responsible for this activity. However, the interpretation can be complicated by the absence of standardized methods to measure anti-SARS-CoV-2 antibodies and to perform neutralizing assays. The frequent mutations of the virus can also affect the neutralizing activity of the antibodies produced against another strain of the virus.

## 5. Conclusions

This study validated the use of a 10-µL self-collected DBS to quantify the level of anti-S antibodies using an automated immunoassay. It can be as reliable as a serum/plasma sample to follow the evolution of the level of neutralizing antibodies against the SARS-CoV-2. This approach would simplify frequent and/or large-scale testing that might be required to evaluate the protection against the SARS-CoV-2 virus in populations after a certain amount of time and help determine whether a booster dose of vaccine is needed.

## Figures and Tables

**Table 1:** **Evaluation of the protocol used to quantify anti-SARS-CoV-2 S antibodies from DBS.** Controls were prepared with different levels of anti-S antibodies, and multiple DBS were spotted and analyzed at different post-sampling times. Robustness of the measures from DBS were evaluated by repeatability and intermediate fidelity experiments after extraction of one 10 µL spot. Five experiments were performed over two weeks by two different analysts. The analyzer was calibrated on day one and day eight, and the positive and negative controls were analyzed with each experiment with satisfactory results.

Plasma Values (U/mL)	DBS Results	Day 1	Day 2	Day 5	Day 8	Day 13	Day 13/ Day 1 (%)
C1: 14.8	C1	0.477	0.426	<0.400	<0.400	<0.400	
		0.439	0.414	0.431	<0.400	<0.400	
		0.417	0.416	<0.400			
		0.445	0.453	0.415			
	**mean**	**0.445**	**0.427**	**0.411**	**<0.400**	**<0.400**	ND
	CV%	5.6%	4.2%	ND	ND	ND	
C3: 129.1	C3	2.99	3.21	2.84	2.76	2.85	
		3.17	3.32	2.89	2.89	2.84	
		3.15	3.18	2.94			
		3.14	3.22	2.92			
	**mean**	**3.11**	**3.233**	**2.898**	**2.825**	**2.845**	**91.4%**
	CV%	2.7%	1.9%	1.5%	3.3%	3.2%	
C4: 59.8	C4	1.680	1.63	1.52	1.66	1.49	
		1.560	1.64	1.53	1.47	1.46	
		1.600	1.59	1.44			
		1.650	1.58	1.55			
	**mean**	**1.623**	**1.610**	**1.510**	**1.565**	**1.475**	**90.9%**
	CV%	3.3%	1.8%	3.2%	8.6%	1.4%	

**Table 2:** **Anti-S antibodies were detected in plasma and DBS following the first phase of the vaccination (Comirnaty vaccine dose 1).** Anti-S immunoglobulins were quantified with the Elecsys anti-SARS-CoV-2 S immunoassay in plasma and in 150 µL buffer after elution from a 10 µL DBS for each volunteer every two weeks after vaccination before administration of the second dose.

	Test before Vaccination		2 Weeks after First Dose (Pfizer)		Fold Plasma/Capitainer	4 Weeks after First Dose		Fold Plasma/Capitainer	4 Weeks after First Dose		Fold Plasma/Capitainer
CODE	Plasma	DBS	Plasma	DBS		Plasma	DBS		Plasma	DBS	
MB-126	<0.4	<0.4	3.52	<0.4		22.15	0.441	50.2	26.16	0.54	48.4
MB-127	<0.4	<0.4	30.9	<0.4		80.14	1.03	77.8	81.77	1.55	52.8
MB-128	<0.4	<0.4	146.7	1.99	73.7	183.7	2.04	90.0	144.1	2.8	51.5
MB-129	<0.4	<0.4	2.69	<0.4		49.58	0.922	53.8	44.47	0.793	56.1
MB-130	<0.4	<0.4	14.45	<0.4		29.94	0.466	64.2	21.42	**<0.4**	ND
MB-131	<0.4	<0.4	43.82	0.494	88.7	46.93	0.832	56.4	95.88	2.32	41.3
MB-132	<0.4	<0.4	39.94	<0.4		279.3	5.23	53.4	256.4	4.81	53.3
MB-133	<0.4	<0.4	25.2	<0.4		129.6	2.42	53.6	124.8	2.85	43.8
MB-134	<0.4	<0.4	30.24	0.44	68.7	62.05	1.31	47.4	65.47	1.78	36.8
MB-135	<0.4	<0.4	12.15	<0.4		33.85	**<0.4**	ND	28.94	0.463	62.5
MB-136	<0.4	<0.4	5.86	<0.4		49.88	0.844	59.1	61.65	0.894	69.0
MB-137	<0.4	<0.4	1003	32.27	31.1	930.8	26.53	35.1	793	26.98	29.4
MB-138	<0.4	<0.4	3.21	<0.4		30.6	0.698	43.8	36.3	0.95	38.2
MB-139	<0.4	<0.4	13.25	<0.4		24.92	0.442	56.4	22.76	0.456	49.9

**Table 3:** **Anti-S antibodies were detected in plasma and DBS following the second dose of the Comirnaty vaccine and up to three months later**. Anti-S immunoglobulins were quantified with the Elecsys anti-SARS-CoV-2 S immunoassay in plasma and in 150 µL buffer after elution from a 10 µL DBS for each volunteer.

	1 Week after Dose 2	2 Weeks after Dose 2		Fold Plasma/DBS	3 Weeks after Dose 2	4 Weeks after Dose 2		Fold Plasma/DBS	5 Weeks after Dose 2	6 Weeks after Dose 2		Fold Plasma/ DBS	2 Months after Dose 2		Fold Plasma/ DBS	3 Months after Dose 2		Fold Plasma/ DBS
CODE	DBS	Plasma	DBS		DBS	Plasma	DBS		DBS	Plasma	DBS		Plasma	DBS		Plasma	DBS	
MB-126	67.1	4605	129.7	35.5	85.3	3505	66.4	52.8	56.0		35.7	ND		29.3	ND	1418	22.6	62.7
MB-127	188.9	13,149	304.0	43.3	189.2	8498	160.3	53.0	137.8	7055	125.2	56.3		77.5	ND	4055	66.9	60.6
MB-128	244.5	11,800	321.9	36.7	180.8	7135	164.6	43.3	104.3		101.5	ND	4290	86.9	49.4	3662	64.0	56.4
MB-129	114.7	9765	171.2	57.0	142.8	6900	122.0	56.6	135.1	5830	109.6	53.2	5440	102.5	53.1	3856	63.0	61.3
MB-130	74.1	4988	120.8	41.3	80.4	3230	60.4	53.5	50.8		32.6	ND		32.3	ND	2101	29.4	71.6
MB-131	101.3		103.6	ND	98.2	4541	86.4	52.5	76.9	4258	72.4	58.8	2925	64.1	45.6	2329	40.4	57.7
MB-132	494.3		362.9	ND				ND	139.5	7465	88.5	53.1		86.0	ND	3506	53.6	65.4
MB-133	247.9	9685	256.8	37.7	165.8	6235	129.4	48.2	130.7		96.9	ND	3940	100.4	39.2	4033	77.6	52.0
MB-134	249.5	10,583	283.7	37.3	172.4	7012	150.5	46.6	109.1	5627	126	44.7	4120	102.0	40.4	3639	66.4	54.8
MB-135	126.9	7665	158.2	48.5	97.4	4532	79.8	56.8	72.7	3998	72.0	55.5	2815	54.9	51.3	1845	39.5	46.8
MB-136	246.8	16,785	387.2	43.4	243.4	10,635	205.1	51.9	172.8	7526	147.5	51.0	6732	112.0	60.1	3228	68.2	47.3
MB-137	138.3	7172	176.9	40.5		5465	104.6	52.2	101.2			ND	3353	76.2	44.0	2290	52.3	43.8
MB-138	121.0	7185	169.2	42.5	113	4714	96.2	49.0	80.3	4153	76.6	54.2	3080	71.2	43.3	2491	50.0	49.8
MB-139	95.5	5213	161.8	32.2	98.4	4178	89.4	46.7	77.8		52.3	ND		45.9	ND	2490	55.0	45.3
**Mean**	**179.3**	**9049.4**	**222.0**	**41.3**	**138.9**	**5890.7**	**116.5**	**51.0**	**103.2**	**5738.8**	**87.4**	**53.4**	**4077.2**	**74.4**	**47.4**	**2925**	**53.5**	**55.4**

**Table 4:** Single test of the detection of anti-S antibodies using DBS in other vaccinated volunteers. Anti-S immunoglobulins were quantified with the Elecsys anti-SARS-CoV-2 S immunoassay in plasma and in 150 µL buffer after elution from a 10 µL DBS.

PFIZER VACCINE	7 Weeks after Dose 2			3 Months after Dose 2		
Hospitalized for COVID-19 in November 2020-Final vaccination March 2021 (dose 2)	**Plasma**	**DBS**	Fold Plasma/DBS	**Plasma**	**DBS**	Fold Plasma/DBS
	100 166	1817	55.1	47 710	944.7	50.5
**PFIZER VACCINE** **Final vaccination April 2021**	**5 Weeks after Dose 2**			**3 Months after Dose 2**		
	**Plasma**	**DBS**	Fold Plasma/DBS	**Plasma**	**DBS**	Fold Plasma/DBS
	4987	91.38	54.6	2854.5	50.24	56.8
**ASTRAZENECA VACCINE** **Final vaccination June 2021**	**3 Weeks after Dose 2**	**5 Weeks after Dose 2**				
	**DBS**	**DBS**				
	**45.3**	**37.94**				
**ASTRAZENECA VACCINE** **Final vaccination July 2021**	**1 Day after Dose 2**	**3 Weeks after Dose 2**				
	**DBS**	**DBS**				
	0.822	88.24				
**JANSSEN VACCINE** **Final vaccination May 2021**	**7 Weeks after the Dose**					
	**DBS**					
	1.96					

**Table 5:** Single test of the detection of anti-S antibodies using DBS in a cohort of 200 volunteers from Stockholm without information on their COVID-19 or SARS-CoV-2 vaccination status. Anti-S immunoglobulins were quantified with the Elecsys anti-SARS-CoV-2 S immunoassay in plasma and in 150 µL buffer after elution from a 10 µL DBS. Differences between plasma and DBS results in the 200 samples.

Sex	Age	Sample Number	Plasma	DBS	Fold Change Plasma/DBS
F	85	4	<0.4	<0.4	ND
M	32	6	<0.4	<0.4	ND
M	39	7	<0.4	<0.4	ND
F	76	38	<0.4	<0.4	ND
F	18	12	<0.4	<0.4	ND
F	10	39	<0.4	<0.4	ND
F	15	20	<0.4	<0.4	ND
M	48	40	<0.4	<0.4	ND
M	33	23	<0.4	<0.4	ND
M	13	57	<0.4	<0.4	ND
M	77	60	<0.4	<0.4	ND
M	73	70	<0.4	<0.4	ND
F	59	74	<0.4	<0.4	ND
M	19	121	<0.4	<0.4	ND
M	77	168	<0.4	<0.4	ND
F	33	37	<0.4	<0.4	ND
?	?	64	<0.4	<0.4	ND
F	26	79	<0.4	<0.4	ND
F	7	88	<0.4	<0.4	ND
M	61	90	<0.4	<0.4	ND
F	15	98	<0.4	<0.4	ND
F	15	159	<0.4	<0.4	ND
M	27	161	<0.4	<0.4	ND
F	48	172	<0.4	<0.4	ND
F	18	199	<0.4	<0.4	ND
M	33	103	0.41	<0.4	ND
F	30	102	1.1	<0.4	ND
F	41	165	1.8	<0.4	ND
M	72	95	1.9	<0.4	ND
F	34	51	6.0	<0.4	ND
M	88	36	6.1	<0.4	ND
M	69	50	6.3	<0.4	ND
F	38	134	7.2	<0.4	ND
F	33	133	12.2	<0.4	ND
F	73	96	13.5	0.46	29.0
M	73	124	16.5	<0.4	ND
M	60	191	18.3	0.48	38.6
F	31	116	18.4	<0.4	ND
M	39	181	18.7	0.65	28.7
F	86	108	19.1	<0.4	ND
F	79	160	19.4	0.65	30.1
F	89	128	25.0	<0.4	ND
F	79	80	25.3	0.62	40.7
F	78	132	32.0	<0.4	ND
F	40	99	32.7	0.81	40.6
F	34	114	33.4	<0.4	ND
F	48	152	34.7	1.06	32.7
M	94	197	39.0	1.05	37.2
M	46	187	40.3	1.42	28.4
F	75	61	44.0	0.99	44.6
M	46	97	48.2	1.48	32.6
F	97	10	53.7	2.10	25.6
M	74	143	55.8	1.36	41.1
F	52	101	61.8	1.49	41.5
F	50	94	64.9	2.03	32.0
M	79	56	66.9	2.22	30.1
F	21	3	72.6	1.84	39.4
F	32	52	75.8	1.99	38.1
M	44	142	80.0	0.92	86.6
M	81	140	80.1	0.48	168.0
F	69	126	92.7	2.62	35.4
F	34	22	110	1.87	58.9
M	31	100	117	2.02	57.7
F	68	146	120	2.99	40.0
F	48	145	122	1.94	63.0
F	11	138	126	<0.4	ND
F	82	2	128	3.56	36.0
F	45	87	129.2	3.98	32.5
M	55	13	138.2	4.86	28.4
M	90	125	143	4.11	34.8
M	54	75	148.5	4.96	29.9
F	81	18	150.1	1.97	76.2
F	87	137	156.5	1.8	86.9
F	62	48	157	8.74	18.0
F	36	171	158.1	2.78	56.9
F	41	164	160.9	4.28	37.6
F	83	9	161.9	3.94	41.1
M	18	67	176.6	4.17	42.4
F	85	163	183.6	5.09	36.1
M	81	89	195.5	2.89	67.6
M	66	58	205.7	5.56	37.0
F	66	104	213.4	5.57	38.3
M	38	54	226.9	5.63	40.3
M	28	55	229.4	2.27	101.1
F	34	107	241.2	5.72	42.2
F	39	49	273	13.43	20.3
M	75	43	279	16.24	17.2
M	89	34	368	11.03	33.4
F	28	17	418	7.45	56.1
F	74	11	452	8.44	53.6
F	81	112	454	7.12	63.8
F	13	30	484	7.85	61.7
F	88	35	486	13.25	36.7
F	39	27	495	9.05	54.7
M	87	26	515	7.72	66.7
F	70	85	536	8.49	63.1
M	75	83	556	10.53	52.8
F	68	44	558	13.3	42.0
M	74	82	575	9.74	59.0
M	47	93	663	13.09	50.6
M	86	117	664	10.47	63.4
F	71	72	727	9.39	77.4
M	52	148	820	11.35	72.2
F	29	65	827	14.29	57.9
F	74	42	842	16.05	52.5
F	84	92	863	16.38	52.7
F	75	81	889	13.44	66.1
M	72	186	937	21.83	42.9
F	89	15	1081	21.64	50.0
M	86	122	1104	21.38	51.6
F	89	111	1147	22.31	51.4
M	13	8	1166	45.84	25.4
F	83	162	1254	24.79	50.6
F	41	175	1259	22.51	55.9
F	67	115	1295	24.45	53.0
F	57	200	1396	24.51	57.0
M	22	130	1398	9.26	151.0
M	25	156	1436	23.78	60.4
F	32	178	1514	24.38	62.1
M	57	157	1618	27.62	58.6
F	60	123	1621	29.58	54.8
F	70	105	1740	33.85	51.4
F	92	177	1762	32.11	54.9
F	91	141	1766	32.74	53.9
M	33	131	1768	44.67	39.6
F	70	113	1788	33.44	53.5
M	63	127	1806	32.82	55.0
M	63	71	1837	25.63	71.7
F	70	106	1884	36.34	51.8
F	83	68	1910	30.45	62.7
M	22	77	1961	27.44	71.5
F	56	120	2038	29.21	69.8
M	46	45	2281	92.63	24.6
F	87	5	2328	45.56	51.1
M	49	47	2331	112.6	20.7
F	79	14	2375	37.23	63.8
M	69	144	2716	68.05	39.9
M	54	147	2722	39.05	69.7
F	49	32	2990	51.63	57.9
M	60	29	3007	47.19	63.7
M	63	189	3075	45.94	66.9
F	53	169	3095	54.46	56.8
M	74	153	3107	54.19	57.3
F	50	78	3163	49.34	64.1
F	43	110	3170	51.24	61.9
F	47	86	3295	57.18	57.6
M	59	198	3448	60.56	56.9
M	59	194	3639	60.27	60.4
M	69	33	3661	62.67	58.4
F	60	69	3960	49.95	79.3
F	76	91	4024	59.12	68.1
M	65	136	4249	70.44	60.3
M	41	139	4794	88.54	54.1
M	29	84	4798	79.75	60.2
F	68	180	5253	93.19	56.4
F	71	149	6048	89.64	67.5
M	25	174	6450	93.17	69.2
F	19	25	6547	119.6	54.7
F	27	192	6597	111.2	59.3
M	56	188	6747	99.29	68.0
F	71	76	6794	98.5	69.0
F	42	46	7312	1023	7.1
F	38	16	7751	119.4	64.9
F	39	176	7803	172.2	45.3
M	50	53	8630	98.05	88.0
M	42	31	8684	141.7	61.3
M	53	193	8945	150.1	59.6
F	52	118	9036	149.9	60.3
F	23	155	9234	173.3	53.3
F	45	158	9473	172	55.1
M	34	185	9646	147.5	65.4
F	70	41	10,470	1216	8.6
F	81	173	11,910	238.3	50.0
M	63	170	11,960	177	67.6
F	38	184	13,000	201.9	64.4
M	25	195	13,220	189.5	69.8
M	40	135	13,240	223.3	59.3
M	43	62	14,710	180.3	81.6
F	55	59	14,890	211.5	70.4
F	30	154	15,070	227.1	66.4
F	41	150	15,640	199.7	78.3
F	29	151	15,880	248.7	63.9
M	34	24	18,260	675	27.1
F	43	196	18,870	714	26.4
M	38	21	18,950	706	26.8
F	63	167	19,110	794	24.1
F	28	183	19,370	832	23.3
F	43	179	20,040	838	23.9
F	61	109	20,070	639	31.4
F	40	1	20,880	852	24.5
F	83	119	22,870	730	31.3
M	23	182	24,160	818	29.5
M	32	28	31,210	760	41.1
F	48	190	31,990	964	33.2
F	57	166	36,430	1479	24.6
M	52	63	47,210	1126	41.9
M	42	129	59,340	1345	44.1
F	81	73	87,400	2141	40.8
F	53	19	92,820	2896	32.1
F	85	66	98,290	2551	38.5
				**Mean fold-change**	**51.3**
		**Anti-S Ig below the Assay LOD (<0.4)**	**Anti-S Ig between 0.4 and 0.8 U/mL**	**Anti-S Ig (Value ≥0.8 for Plasma or ≥0.4 for DBS)**	
Plasma		25	1	174	
qDBS		41	5	159	
**Discordant plasma-DBS results**					
Plasma ≥0.8 and <0.4 by DBS			15		
Plasma <0.8 and DBS ≥0.4)			0		

## Data Availability

All main data are presented in the manuscript; additional data can be requested directly to the authors by e-mail.
